# Tissue Washing Improves Native Ambient Mass Spectrometry Detection of Membrane Proteins Directly from Tissue

**DOI:** 10.1021/jacs.3c03454

**Published:** 2023-07-17

**Authors:** Emma K. Sisley, Oliver J. Hale, James W. Hughes, Helen J. Cooper

**Affiliations:** School of Biosciences, University of Birmingham, Birmingham B15 2TT, U.K.

## Abstract

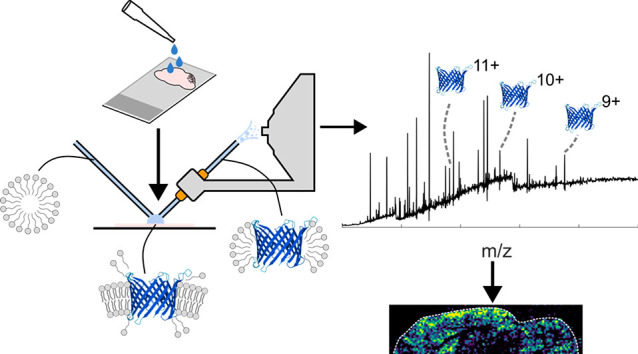

Native ambient mass spectrometry enables the *in situ* analysis of proteins and their complexes directly from tissue, providing both structural and spatial information. Until recently, the approach was applied exclusively to the analysis of soluble proteins; however, there is a drive for new techniques that enable analysis of membrane proteins. Here we demonstrate native ambient mass spectrometry of membrane proteins, including β-barrel and α-helical (single and multipass) integral membrane proteins and membrane-associated proteins incorporating lipid anchors, by integration of a simple washing protocol to remove soluble proteins. Mass spectrometry imaging revealed that washing did not disrupt the spatial distributions of the membrane and membrane-associated proteins. Some delocalization of the remaining soluble proteins was observed.

Native ambient mass spectrometry (NAMS) combines native mass spectrometry, in which inter- and intramolecular non-covalent interactions present in solution are maintained in the gas phase, and ambient mass spectrometry, in which substrates such as thin tissue sections are analyzed directly with little or no sample preparation.^1−3^ NAMS provides information about the structure of proteins and their spatial distribution in a single experiment. To date, NAMS has focused primarily on soluble proteins and their assemblies and complexes.^4−6^

Membrane proteins constitute around a third of the proteome but around two-thirds of therapeutic targets.^7,8^ Moreover, a number of measures of research progress indicate that knowledge of membrane proteins lags behind that of soluble proteins by a number of decades.^9^ We recently demonstrated that NAMS may be extended to membrane proteins using the example of highly abundant aquaporin-0 (Aqp-0). We showed that Aqp-0 can be observed directly from eye lens tissue by use of a sampling solvent containing the detergent tetraethylene glycol monooctyl ether (C8E4) at a concentration greater than the critical micelle concentration (CMC).^10^ Despite these results, empirical observations in our laboratory suggest that the use of C8E4 at concentrations greater than the CMC alone is not sufficient to allow detection of membrane proteins more broadly, e.g., those in other tissue types or in lower abundance. Here, we demonstrate that inclusion of a washing step prior to NAMS analysis enables the detection of integral membrane and membrane-associated proteins from thin sections of the rat brain and kidney. The use of washing protocols to remove lipids is well-established in mass spectrometry imaging.^11,12^ The aim here was not to remove lipids, as they are necessary for stabilization of membrane proteins, but to remove more soluble proteins, thereby reducing any ion suppression effects, i.e., ionization of soluble proteins at the expense of less abundant membrane proteins. Washing may also remove other cytosolic materials and salts, again reducing any ion suppression effects.

Washing was achieved by pipetting the wash solvent onto the tissue section, such that the entire section was covered, followed by inversion of the slide to drain off the wash solvent and drying in a vacuum desiccator. Both MS-grade water and 200 mM aqueous ammonium acetate were investigated as potential wash solvents. Tissue washing with water has previously been applied in the analysis of proteolipid protein (PLP) from brain tissue by matrix-assisted laser desorption/ionization (MALDI) mass spectrometry.^13^ In our hands, washing with water resulted in visible disruption of the thaw-mounted tissue and therefore loss of spatial information. The ammonium acetate wash, however, left the tissue intact (see Figure S1). Nanospray desorption electrospray ionization (nano-DESI)^6,14,15^ sampling was performed as described previously, i.e., using aqueous ammonium acetate solvent containing C8E4 micelles to dissolve membrane proteins.^10^ Micelle-encapsulated membrane proteins were ionized and introduced into the mass spectrometer. Control experiments were performed in which unwashed tissue was sampled with 0.5 × CMC and 2 × CMC detergent and washed tissue was sampled with 0.5 × CMC detergent. Full experimental details are given in the Supporting Information.

Figure 1a,b shows summed nano-DESI mass spectra obtained from the cortex region of the brain from unwashed and washed tissue using a sampling solvent system comprising 200 mM ammonium acetate + 2 × CMC C8E4 and a source compensation value (SCV) of 3% (see Figure S2 for brain anatomy). The proteins observed in the mass spectrum from unwashed tissue are similar to those observed when using a lower concentration of detergent (0.5 × CMC) in the sampling solvent (see Figure S3). All proteins labeled in the mass spectra from unwashed tissue have been identified in previous work^4,5^ and are assigned here on the basis of intact mass. The mass spectrum from washed tissue differs markedly from that of unwashed tissue. Newly detected proteins were identified by top-down fragmentation followed by protein database searching (for details, see Table S2)*.*Table S3 provides a summary of all proteins identified together with known abundance and spatial distributions where available in the literature. The proteins identified include the 25.8 kDa ras-related protein Rab-3A (Rab3A) and the 21.9 kDa brain acid soluble protein 1 (BASP1) (Figures S4 and S5). Crucially, the washing protocol together with use of detergent at 2 × CMC resulted in detection of the membrane protein voltage-dependent anion channel 1 (VDAC1) (30.6 kDa; see Figure 2a). VDAC1 is a β-barrel membrane protein which is located in the outer membrane of mitochondria and controls the transport of cations and respiratory substrates across the membrane.^16^

**Figure 1:**
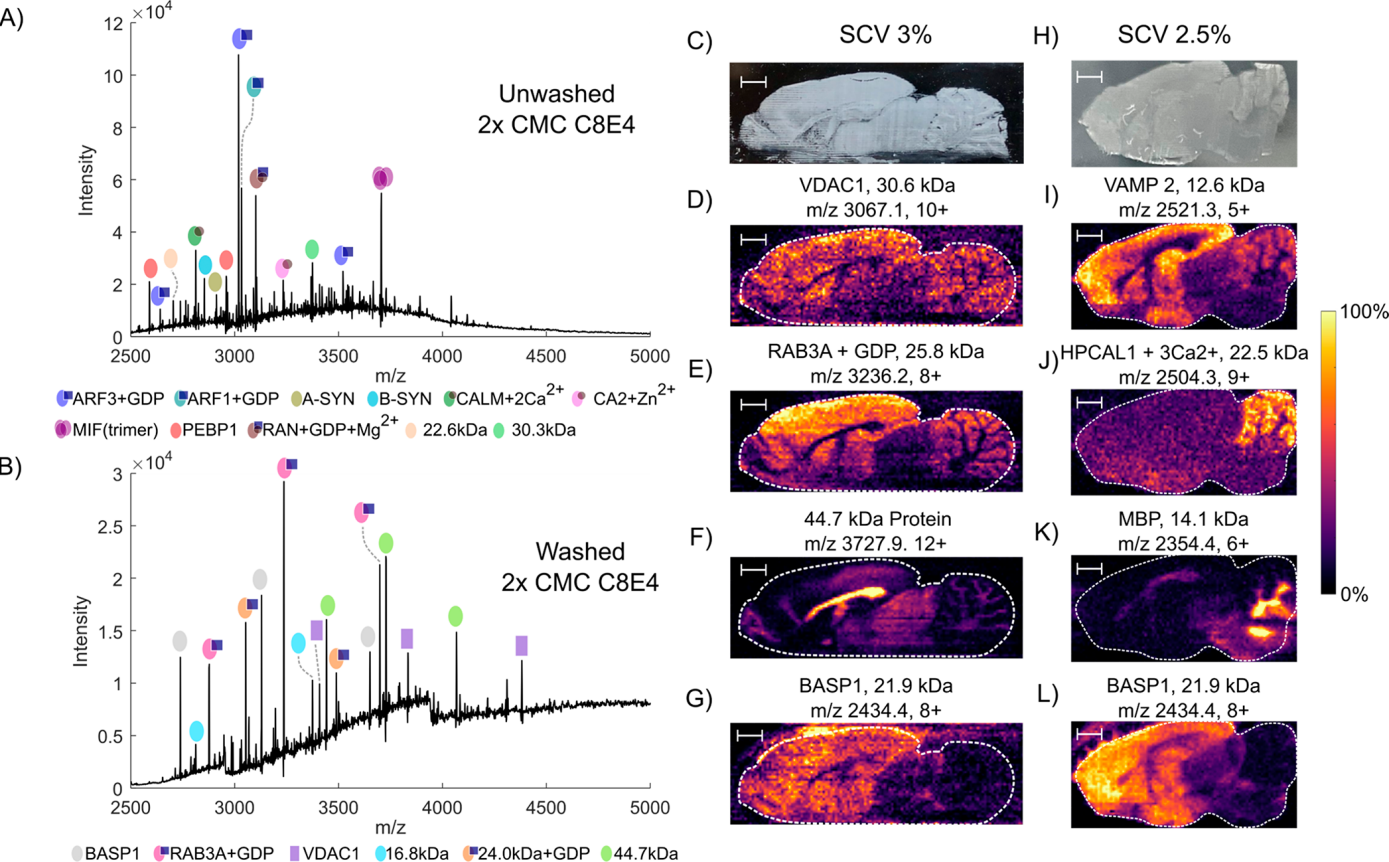
Summed nano-DESI mass spectra obtained following sampling of the cortex region in (A) unwashed and (B) washed brain tissue sections. Nano-DESI sampling solvent contained 2 × CMC C8E4 detergent. (C) Photograph of a washed brain section after sampling and (D–G) corresponding single-charge-state ion images obtained using a source compensation value (SCV) of 3%. (H) Photograph of a washed brain section after sampling and (J–L) corresponding single-charge-state ion images obtained with an SCV of 2.5%. Scale bars denote 2 mm.

**Figure 2:**
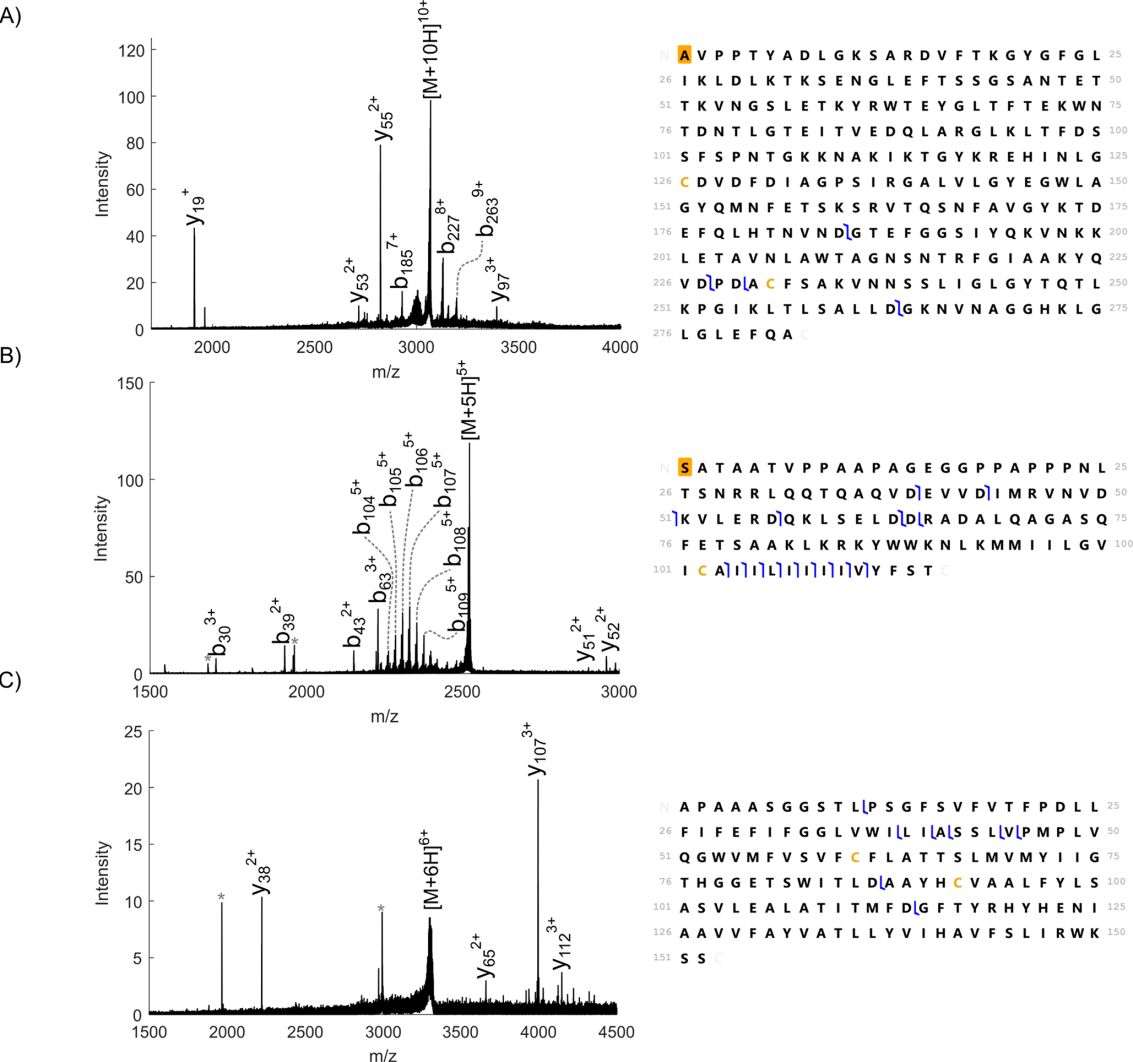
Top-down fragmentation of membrane proteins sampled directly from tissue using nano-DESI. (A) HCD MS^2^ of 10+ ions of N-acetylated VDAC1 (*m*/*z* 3067.6 ± 7.5, NCE 55%). (B) HCD MS^2^ of 5+ ions of N-acetylated VAMP2 (*m*/*z* 2521.3 ± 5, NCE 36%). (C) HCD MS^2^ of 6+ ions of MAL (*m*/*z* 3311.9 ± 7.5, NCE 35%). N-terminal acetylation is marked by an orange box. * indicates noise peaks.

By tuning the SCV (an “uphill” voltage in the source optics which reduces an ion’s kinetic energy to help transmission through the flatapole), it is possible to preferentially transmit ions over different *m*/*z* ranges^1,4,17^ (see Figure S6). At an SCV of 2.5%, vesicle-associated membrane protein 2 (VAMP2) with N-terminal acetylation (Figure 2b) was detected. VAMP2 (12.6 kDa) is a single-pass membrane protein found in synaptic vesicles. It is involved in the docking of the vesicle with the plasma membrane by formation of the SNARE complex.^18^ Other newly detected proteins at an SCV of 2.5% were hippocalcin-like protein 1 (HPCAL1) with N-terminal myristoylation and non-covalent attachment of three Ca^2+^ ions (22.5 kDa) and the 14.1 kDa short isoform of myelin basic protein (MBP) (Figures S7 and S8).

Figure 1c shows a photograph of a section of brain tissue (BE002350-13/An8) after nano-DESI sampling. Figure 1d–g shows the corresponding ion images obtained with an SCV of 3% (higher *m*/*z*). Figure 1h shows a photograph of a brain section (BE002350-13/An2), with Figure 1i–l showing ion images obtained using an SCV of 2.5% (lower *m*/*z*). The two membrane proteins (VDAC1 and VAMP2) display distinct distributions. VDAC1 is distributed throughout the brain but is absent in the corpus callosum and midbrain. VAMP2 also displayed a strong signal in the cortex, hippocampus, and thalamus and a weaker signal in the gray matter of the cerebellum. Both the distributions of VDAC1 and VAMP2 are in agreement with previous immunohistochemical studies^19,20^ and confirm that the spatial distribution of the membrane proteins were not disrupted by the washing procedure.

In addition to integral membrane proteins, tissue washing enabled the detection of membrane-associated proteins. Rab3A (Figures 1e and S4) and HPCAL1 (Figures 1j and S7) were observed to have distinct spatial distributions (also see Figure S9). Rab3A is abundant in the cortex, basal ganglia, thalamus, and gray matter of the cerebellum and was observed to be modified with hydrophobic *S*-geranylgeranyl groups on the two cysteine residues at its C-terminus.^21^ Rab3A is involved in vesicle docking and tethering and has a similar distribution to VAMP2, which is also involved in this process.^22^ Importantly, membrane localization is dependent on the geranylgeranyl lipid anchors.^23^ In addition, Rab3A was observed to have GDP non-covalently bound, suggesting that washing does not necessarily disrupt non-covalent interactions. HPCAL1 was observed primarily in the cerebellum, in agreement with *in situ* hybridization experiments.^24^ HPCAL1 was observed to be modified by myristoylation, a known membrane lipid anchor, at the N-terminus and bound to three Ca^2+^ ions. The non-covalent binding of the three Ca^2+^ ions induces a conformational change in the protein, exposing the myristoyl anchor and enabling the protein to attach to a membrane.^25^ The observation of the Ca^2+^ ions further suggests that washing enables imaging of membrane-associated proteins without disrupting their non-covalent interactions. MBP short isoform was observed in the white matter of the cerebellum (Figure 1k). MBP is essential for compact myelin membrane stacking and can undergo partial membrane insertion.^26^ Lastly, an unidentified 44.7 kDa protein was observed with a highly distinctive spatial distribution, localized to the corpus callosum (Figure 1f). Top-down fragmentation did not allow identification of this protein, but its sharp ion image suggests that it is a membrane or membrane-associated protein.

BASP1 was observed at both SCVs (Figure 1g,l) and, importantly, was observed from washed tissue following sampling with solvent containing 0.5 × CMC detergent (Figure S6), suggesting that, despite the myristoylation at the N-terminus, it is more soluble than other proteins detected. The increased solubility is due to a highly acidic region within the protein.^27^ Consequently, some delocalization of the protein is observed in both images. Protein signal was detected from the glass slide adjacent to the tissue section, and the distribution within the tissue is less focused compared to, e.g., VAMP2 and Rab3A. GDP-bound ARF1 was observed in the nano-DESI mass spectra obtained following washing (Figures 1a and S6). ARF1 is a soluble protein and is also detected in the absence of washing and at lower detergent concentrations.^5^ Our findings show that the spatial distribution of this protein was disrupted as a result of washing (Figure S10).

The workflow was also applied to sections of rat kidney. Nano-DESI mass spectra obtained from the renal cortex are shown in Figure S11. Again, the unwashed tissue yielded a mass spectrum heavily populated with peaks corresponding to soluble proteins while the washed tissue yielded a range of newly detected signals. *In situ* top-down fragmentation resulted in the identification of the membrane protein VDAC1 (Figure S12). Nano-DESI mass spectra obtained from the cortex, medulla, and renal pelvis regions of washed tissue sections are shown in Figure S13. VDAC1 was particularly abundant in the cortex and medulla of the kidney. Two further membrane proteins, myelin and lymphocyte protein (MAL) (16.5 kDa) and cytochrome *b*_5_ (CYB5A) (15.3 kDa), were identified from the washed tissue (Figures 2C and S14). MAL is a tetraspan membrane protein involved in formation and stabilization of lipid rafts^28^ and was observed here in the renal pelvis. CYB5A binds heme non-covalently and has a C-terminal transmembrane helix and was observed here in the cortex. Interestingly, the protein was observed without its prosthetic group, suggesting that in this case washing removed the soluble heme. Lastly, actin 1, likely bound to ADP (42.1 kDa) due to the characteristic mass shift observed in the fragmentation data, was also identified throughout the kidney (Figure S15).

In conclusion, our results show that tissue washing with 200 mM ammonium acetate prior to nano-DESI sampling reduces or eliminates abundant signals from soluble proteins, enabling the detection of integral membrane and membrane-associated proteins directly from tissue sections. Membrane proteins were observed only when using 2 × CMC detergent in the sampling solvent. The ion images show that the spatial distributions of the membrane (and membrane-associated) proteins are not disrupted by washing. Some delocalization was observed for the more soluble proteins (BASP1 and ARF1). Future work will focus on trialing alternative detergents, with the aim of increasing the depth of membrane protein coverage.

## Data Availability

Supplementary data supporting this research are openly available from DOI: https://doi.org/10.25500/edata.bham.00000962.

## Supporting Information Available

Full experimental details, photos of tissue after washing, representative MS spectra, MS^*n*^ spectra and sequence coverages, and additional ion images (PDF)
