# Supplementary material for: Tissue Washing Improves Native Ambient Mass Spectrometry Detection of Membrane Proteins Directly from Tissue
Source: J Am Chem Soc. 2023 Jul 17;145(29):15658–62. doi: 10.1021/jacs.3c03454 (PMC10375469; doi:10.1021/jacs.3c03454)
Supplement: Supplementary file 1 — ja3c03454_si_001.pdf [file ja3c03454_si_001.pdf]

Supporting information for:

Tissue Washing Improves Native Ambient Mass Spectrometry Detection of Membrane Proteins Directly from Tissue

Emma K. Sisley[1], Oliver J. Hale[1], James W. Hughes[1] and Helen J. Cooper[1]

[1.] School of Biosciences, University of Birmingham, Birmingham, B15 2TT, UK

Contents

Experimental methods

Table S1: Protocol for H&E staining of tissue

Figure S1: Photographs of tissue after different washes

Figure S2: Schematic showing the regions of the rat brain.

Figure S3: Representative nano-DESI mass spectra obtained from the cortex of the brain using a source compensation value (SCV) of 3%

Table S2: Details of top-down fragmentation of protein ions

Table S3: Summary of proteins identified from washed and unwashed brain tissue

Figure S4: Nano-DESI HCD MS^2 mass spectrum of m/z 3236.2 8+ ions of RAB3A

Figure S5: Nano-DESI HCD MS^2 mass spectrum of m/z 2738.5 8+ ions of BASP1

Figure S6: Representative nano-DESI mass spectra from the cortex of the brain using a source compensation value (SCV) of 2.5%

Figure S7: Nano-DESI HCD MS^2 mass spectrum of m/z 2504.3 9+ ions of hippocalcin-like protein 1

Figure S8: Nano-DESI HCD MS^2 mass spectrum of m/z 2354.6 6+ ions of MBP short form

Figure S9: Single charge state ion images of Rab3a and HPCAL1

Figure S10: Ion images of [ARF1 + GDP], showing the disruption of spatial distribution in washed tissue compared to unwashed

Figure S11: Representative nano-DESI mass spectra obtained from the cortex region of the kidney from washed and unwashed tissue

Figure S12: Nano-DESI HCD MS^2 mass spectrum of m/z 3067.6 10+ ions of VDAC1 (HCD 55% NCE)

Figure S13: Representative nano-DESI mass spectra obtained from cortex, medulla, and renal pelvis regions of washed rat kidney section sampled using 2x CMC C8E4.

Figure S14: Nano-DESI HCD MS^2 mass spectrum of m/z 2545.2 6+ ions of cytochrome B5

Figure S15: Nano-DESI HCD MS^2 mass spectrum of m/z 3510.2 12+ ions of actin 1 with ADP bound (HCD 51% NCE).

References

Experimental

Materials

Brain and kidney tissue from two control vehicle-dosed (0.5% hydroxypropyl methylcellulose (HPMC) and 0.1% tween 80 in water) adult male Han-Wistar rats (denoted BE002350-13/An2 and BE002350-13/An8) were the kind gift of Prof. Richard Goodwin, AstraZeneca (i.e., extraneous control tissue from a drug dosing experiment). Animals were euthanized 6 hrs post dose, and dissection was performed by trained AstraZeneca staff (project license PP7736793, procedure number 3). Brains and kidneys were snap-frozen in isopentane over dry ice and stored at −80 °C. Tissues were sectioned in the sagittal plane to 10 µm thickness with a CM1810 Cryostat ((Leica Microsystems, Wetzlar, Germany) and thaw mounted onto glass slides at -24 ^{o}C. Tissue sections were stored at -80 ^{o}C until analysis.

MS-grade water was purchased from Fisher Scientific (Loughborough, UK), C8E4 detergent was purchased from Sigma-Aldrich (Gillingham, UK), and HPLC grade ammonium acetate was purchased from J. T. Baker (Deventer, Netherlands). Nitrogen and helium supplied to the mass spectrometer were obtained from BOC (Guilford, U.K.). Harris hematoxylin, acid alcohol, industrial denatured alcohol, Scott's tap water substitute, xylene, and eosin (1% aqueous) were purchased from pfm Medical (Cheshire, U.K.). DPX was purchased from CellPath (Powys, U.K.).

Tissue Washing

The sectioned tissue was allowed to defrost for 10 mins at room temperature before 250-300 µL of 200 mM ammonium acetate or MS-grade water was deposited on top of the tissue, making sure the tissue was fully covered. The washing solvent was left for 60 s before inversion of the slide to drain off the wash solvent. The slide was then placed in a vacuum desiccator for 10 mins to dry completely. The washing and drying process was repeated 3 times. Three washes were found to remove the majority of signals observed prior to washing. After one wash, signals corresponding to soluble proteins could still be detected. Increasing the number of washes above 3 resulted in negligible change in the mass spectra (data not shown).

Nano DESI

Nano-DESI was performed using a home-built ion source described previously[1, 2]. Briefly, the source was mounted to the front of an Orbitrap Eclipse mass spectrometer (Thermo Fisher Scientific, San Jose, CA). The two capillaries were flame-pulled from fused silica tubing (O.D. 275 µm I.D. 75 µm). A 10 mL gastight syringe (Hamilton, Reno, NV) delivered solvent (200 mM aqueous ammonium acetate with either 0.5x CMC C8E4 or 2x CMC C8E4) at 1.8-2 µL/min to maintain a stable solvent bridge between the two capillaries. The high voltage power supply was connected directly to the syringe needle and a potential of 900-1200 V was applied for optimal nanoelectrospray stability.

For nano-DESI images and line scans, an instrument method was written in Xcalibur (V4.3, Thermo) using settings described below. A tissue section mounted onto a glass slide was loaded into the nano-DESI stage. On starting stage movement, contact closure triggered mass spectrometry data acquisition. Each line scan in the nano-DESI ion image was acquired in an automated sequence over the course of approximately 12-16 h, during which the tissue was maintained at room temperature.

The stage moved the tissue section under the nano-DESI probe at a rate of 25 µm/s, with a 200 µm step between each line scan.

Mass Spectrometry

Mass spectrometry data were acquired on an Orbitrap Eclipse Tribrid MS (Thermo), with HMRn option. The instrument was operated in 'Intact Protein' mode and at 'high pressure' (20 mTorr in the IRM). The ion transfer tube was set to a temperature of 275 ^{o}C, the source dissociation value was set to 90 V, and the S-lens RF was set to 120%. The source compensation value (SCV) was set to either 3% or 2.5% as indicated in the main text. Data collected at SCV 3% were acquired in full profile mode (m/z 2500-5000). Data collected at SCV 2.5% were acquired using a 1500 m/z width SIM window centred around m/z 2750. All imaging data were recorded in the Orbitrap analyser at a resolution of 7500 at m/z 200.

Tandem Mass Spectrometry

Top-down MS^2 analysis of the intact proteins was performed by nano-DESI sampling of serial tissue sections. For tandem mass spectrometry experiments, the stage movement was reduced to 2-5 µm/s to maintain signal intensity, and mass spectrometry data acquisition was triggered manually. Precursor ions were isolated in the ion trap using an isolation window between 10 and 15 m/z and fragmented by use of a normalised collision energy (NCE) between 35 and 55% (see *Table S2*). All data were recorded in the Orbitrap mass analyser at a resolution of 240,000 at m/z 200.

Data Analysis

Nano-DESI images were produced by acquiring line scans in sequence. The line scans were converted from Thermo .raw files into a single imzML file using firefly (v3.2.0.23, Prosolia, Inc). The bin size was set to 0.133 mins to create 200 µm pixels. Ion images were produced in MSI reader using a 0.2 m/z window across the protein signal apex and first order linear interpolation. No normalisation was applied.

Proteins were identified from MS^2 data using ProSight 4.1 (Thermo). Data were summed in Freestyle (Thermo) and then imported into Prosight. Fragmentation data was searched against the *Rattus norvegicus* proteome (downloaded from Proteinacious.net July 2020). Precursor mass tolerance was set between 1-2 kDa to allow for hits containing ligands and modifications, fragment tolerance was set to 20 ppm and minimum fragment match was set to 1. Protein assignments were manually confirmed using Protein Prospector (v6.4.2, https://prospector.ucsf.edu/prospector/mshome.htm , UCSF). All post translational modifications identified here are in agreement with data available from UniProt.

H&E staining

Haemotoxylin and eosin (H&E) staining was performed as described in *Table S1*. Thaw mounted tissue sections were sequentially submerged in solvent baths containing the described solvent and for the time listed. Where a repeat submersion was required, a fresh bath of solvent was used. Tissue was mounted in DPX and a coverslip was applied before optical imaging.

Table S1: Protocol for H&E staining of tissue

Solvent Bath	Time (min)	Repeats
Water	2	1
Haemotoxylin Harris	4	0
Water	2	1
Acid Alcohol	0.5	0
Water	2	1
Scott's Tap Water Substitute	0.5	0
Water	2	1
Eosin	1	0
Water	2	1
Industrial Denatured Alcohol	2	3
Xylene	2	2

A) B) C)

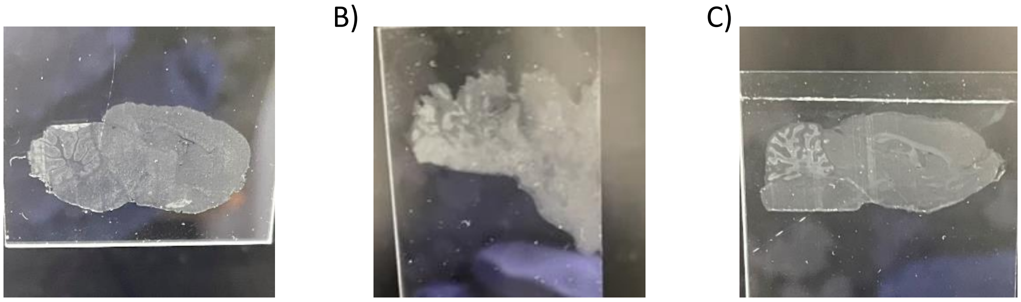

Unwashed

MS-grade Water (after 2 washes)

200 mM ammonium acetate (after 3 washes)

Figure S1: Photograph of A) unwashed section of brain tissue, B) section of brain tissue after 2 washes with MS grade water and C) section of brain tissue after 3 washes with 200 mM ammonium acetate.

A) B)

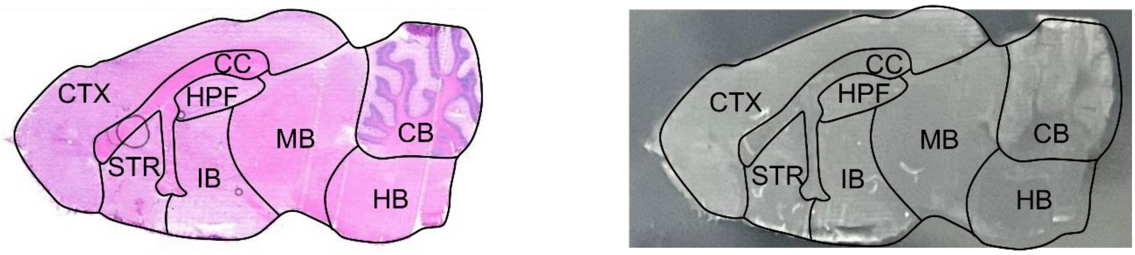

Figure S2: Schematic showing the regions of the rat brain. A) H&E stained tissue section and B) a photograph of the same section (before staining). Labels have been defined using the brain atlas (atlas.brain-map.org). Cortex (CTX), corpus callosum (CC), hippocampal formation (HPF), striatum (STR), interbrain (IB), midbrain (MB), hindbrain (HB), cerebellum (CB).

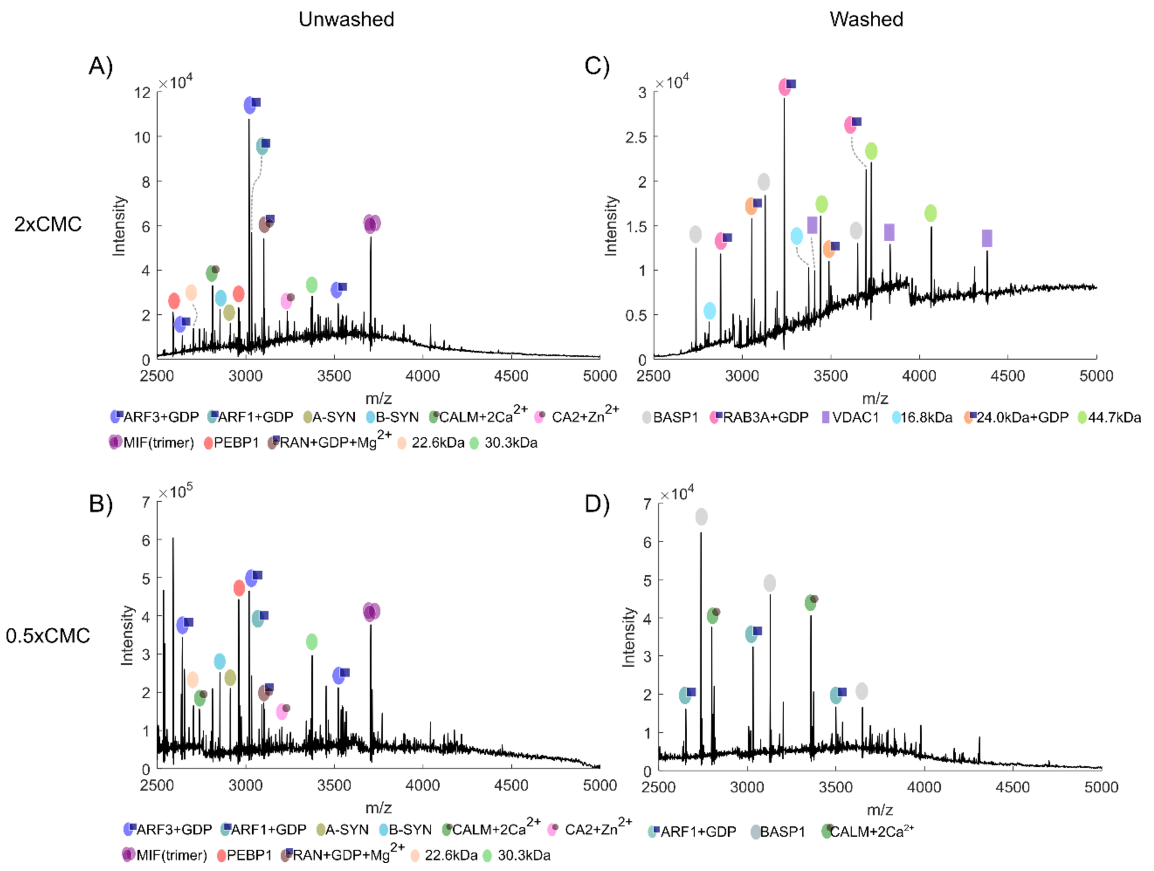

Figure S3: Representative nano-DESI mass spectra obtained from the cortex of the brain obtained with SCV of 3% and in full scan mode m/z 2500-5000: A) unwashed tissue sampled with 200 mM ammonium acetate containing 2x CMC C8E4, B) unwashed tissue sampled with 200 mM containing 0.5x CMC C8E4, C) washed tissue sampled with 200 mM ammonium acetate containing 2x CMC C8E4 and D) washed tissue sampled with 200 mM ammonium acetate containing 0.5x CMC C8E4. Proteins were identified either based on measured intact mass and prior knowledge from our previous publications, or top-down MS/MS as documented below. Averaged mass spectra were achieved by recording three nano-DESI line scans. These were imported into MSI reader as an IMZL file where the ROI tool was used to export an averaged mass spectrum of the cortex.

Table S2: Details of top-down fragmentation of protein ions:

m/z	Charge state	HCD NCE (%)	Protein ID
3067.6	10	55	VDAC1
2521.3	5	36	VAMP2
3236.2	8	43	RAB3A + GDP
2738.5	8	40	BASP1
2504.3	9	48	HPCAL1
2354.6	6	44	MBP (short isoform)
3311.9	5	35	MAL
3510.2	12	51	Actin 1 + ADP
2545.2	6	40	Cytochrome B5

Table S3: Summary of proteins detected from A) unwashed brain tissue and B) washed brain tissue. [a]As described in UniProt (https://www.uniprot.org/). [b]As described in Human Protein Atlas (proteinatlas.org). *Denotes proteins observed only after washing, but with sampling solvents containing either 0.5x CMC or 2x CMC of detergent. (All other proteins detected in washed tissue were only observed with sampling solvent containing 2x CMC detergent).
** Denotes proteins observed in both washed and unwashed tissue.

A) Proteins identified in unwashed tissue

Protein	PTMs and ligands detected	Cell location[a]	Tissue specificity	Function	Abundance in brain	Spatial distribution previously determined by nano-DESI[1, 2]	Spatial distributions described in the literature
PEBP1	N-terminal acetylation	Cytoplasm	Many organs, highest levels in liver, kidney, pancreas and brain[b]	Involved in the regulation of cell proliferation and differentiation[3]	No literature data available.	Homogenous throughout the brain	No literature data available.
CALM	N-terminal acetylation , 0-4 Ca^{2+} ions	Cytoplasm	Expressed in most tissues with high expression in tissue and brain[b]	Part of a calcium signal transduction pathway which mediates the control of a large number of enzymes, ion channels, aquaporins and other proteins through calcium-binding[a]	Highly abundant in the central nervous system and it is specially enriched in sites involved in neurotransmission[4]	Ion image not created	N/A
ARF3	N-terminal myristoylation, GDP	Cytoplasm/golgi	Expressed in most human tissues including brain, breast and bladder[5]	Involved in protein trafficking; may modulate vesicle budding and uncoating within the Golgi apparatus[a]	No literature data available.	Cortex, hippocampus, striatum, interbrain, cerebellum	In agreement with gene expression experiment[6]
ARF1	N-terminal myristoylation, GDP	Synapse, golgi apparatus membrane (lipid anchor)	Expressed in most human tissues including brain, breast and bladder[5]	Membrane trafficking and structure in the endoplasmic reticulum–Golgi system[a]	No literature data available.	Cortex, hippocampus, striatum, interbrain, cerebellum	No literature data available.
RAN	N-terminal acetylation, GDP	Cytoplasm/nucleus	Expressed in most tissues including kidney, brain and stomach[7]	Essential for the translocation of RNA and proteins through the nuclear pore complex[a]	No literature data available.	Widely distributed, absent in corpus callosum	No literature data available.
MIF		Cytoplasm	Produced in most tissue types. In the brain, it is expressed in most cells including microglia, astrocytes and neurons[a,8]	Inflammatory mediator[9]	Medium or low expression in all brain regions[b]	Homogenous throughout the brain	In the murine brain, MIF transcripts and protein are mainly present in the cortex, hippocampus, and pituitary gland[9]
SNCB	N-terminal acetylation	Cytoplasm	Brain tissue, mainly in presynaptic terminals[10]	Involved in regulating synaptic function, lipid binding and dopamine neurotransmission[11]	α- and β-synucleins are abundant and concentrated in nerve terminals[12]	Widely distributed, absent in corpus callosum	Appears to be in agreement with in-situ hybridisation experiment, however that experiment does not consider all regions of the brain[13]

SNCA	N-terminal acetylation	Cytoplasm, synapse axon	Brain tissue, predominantly in the neurons of the central nervous system[10]	Regulates synaptic function.[a] Aggregation of this protein in patients with Parkinson's disease[14]	α-Synuclein is abundantly expressed in the nervous system, comprising 1% of total cytosolic protein[15]	Cortex, hippocampus, striatum, interbrain, cerebellum	In agreement with MALDI imaging of peptides[16]
CAH2	N-terminal acetylation	Cytoplasm, cell membrane	Found in a range of tissues, with high abundance in the brain, kidney and digestive tract[b]	Catalyses hydration of CO2[a]	No literature data available.	Most abundant in corpus callosum with weaker signal in midbrain.	CA2 is found in glial cells, which make up a high percentage of the corpus callosum, explaining the observed distribution.[17]
PPIA		Cytoplasm nucleus	Expression in most tissues[b]	Involved in protein folding	No literature data available.	Widely distributed, absent in corpus callosum	No literature data available.
FKBP1A		Cytoplasm	Abundantly expressed in all tissues[18]	Involved in protein folding[18]	Abundantly expressed in all tissues[18]	Hippocampus and striatum	No literature data available.

B) Proteins identified in washed tissue

Protein	PTMs and ligands detected	Cell location[a]	Tissue specificity	Function	Abundance in brain	Experimentally observed spatial distribution	Spatial distributions described in the literature
Rab3a	N-terminal acetylation, x2 geranyl-geranylation, GDP	Cytoplasm / cell membrane (lipid anchor)	Brain, Synaptic vesicles[b,19]	Neurotransmitter release Transport of synaptic vesicles to active zone[a]	Highest expression of RAB3a is in the brain[19]	Cortex, interbrain, striatum, cerebellum, hippocampus. Note very similar distribution to VAMP2, both proteins are involved in the same process.	Immunoblotting shows RAB3A to be present in all regions of the brain with thicker bands for the hippocampus and cortex[20]
VDAC1	N-terminal acetylation	Mitochondrial outer membrane	Expressed in most tissues including brain, kidney and muscle[b]	Ion and small molecules (i.e. ATP/GTP) transporter[21]	Most abundant protein in the outer mitochondrial membrane[21]	Cortex, striatum, cerebellum, lower signal in hippocampus and interbrain	No literature data available.
VAMP2	N-terminal acetylation	Synaptic vesicle membrane	Brain, synaptic vesicles[b]	Involved in neurotransmitter release[a]	Up to 70 copies per SV[22]	Cortex, hippocampus, striatum, interbrain, lower signal in cerebellum	Immunohistochemical staining is in agreement although that experiment was performed on coronal section so cannot judge cerebellum and striatum[23]
HPCAL1	N-terminal myristoylation, 3 Ca^{2+} ions	Membrane (lipid anchor)	Brian, Purkinjie and granule cells[24]	May regulate synaptic plasticity relevant for learning and memory[24]	No literature data available.	Cerebellum	Strong expression in the cerebellum shown by *in-situ* hybridisation exmperiments[25]
MBP	N-terminal acetylation	Myelin membrane (peripheral protein)	Central and peripheral nervous system (myelin sheath)[b]	Maintains the correct structure of myelin[a]	Second most abundant protein in the central nervous system[26]	Cerebellum, hindbrain, lower signal in corpus callosum	MALDI imaging of peptides show distribution of MBP in corpus callosum[27]

CALM**	N-terminal acetylation , 0-4 Ca^{2+} ions	Cytoplasm	Expressed in most tissues with high expression in tissue and brain[b]	Part of a calcium signal transduction pathway which mediates the control of a large number of enzymes, ion channels, aquaporins and other proteins through calcium-binding[a]	Highly abundant in the mammalian central nervous system, especially enriched in sites involved in neurotransmission[4]	Ion image not created	N/A
Arf1**	N-terminal myristoylation, GDP	Synapse, golgi apparatus membrane (lipid anchor)	Expressed in most human tissues including brain, breast and bladder[5]	Membrane trafficking and structure in the endoplasmic reticulum–Golgi system[a]	No literature data available.	Spatial distribution disrupted in washing step	N/A
BASP1*	N-terminal myristoylation	Cell membrane (lipid anchor)	Brain, expressed predominantly in neurons[28]	Implicated in neurite outgrowth, maturation of the actin cytoskeleton, and organization of the plasma membrane[28]	0.4–0.8% of the total brain protein[29]	Spatial distribution slightly disrupted in washing step - Cortex, striatum, interbrain, hippocampus	Good agreement - Wide distribution of BASP1 in the brain, particularly in the synapse region has been shown using immunohistochemistry[30]

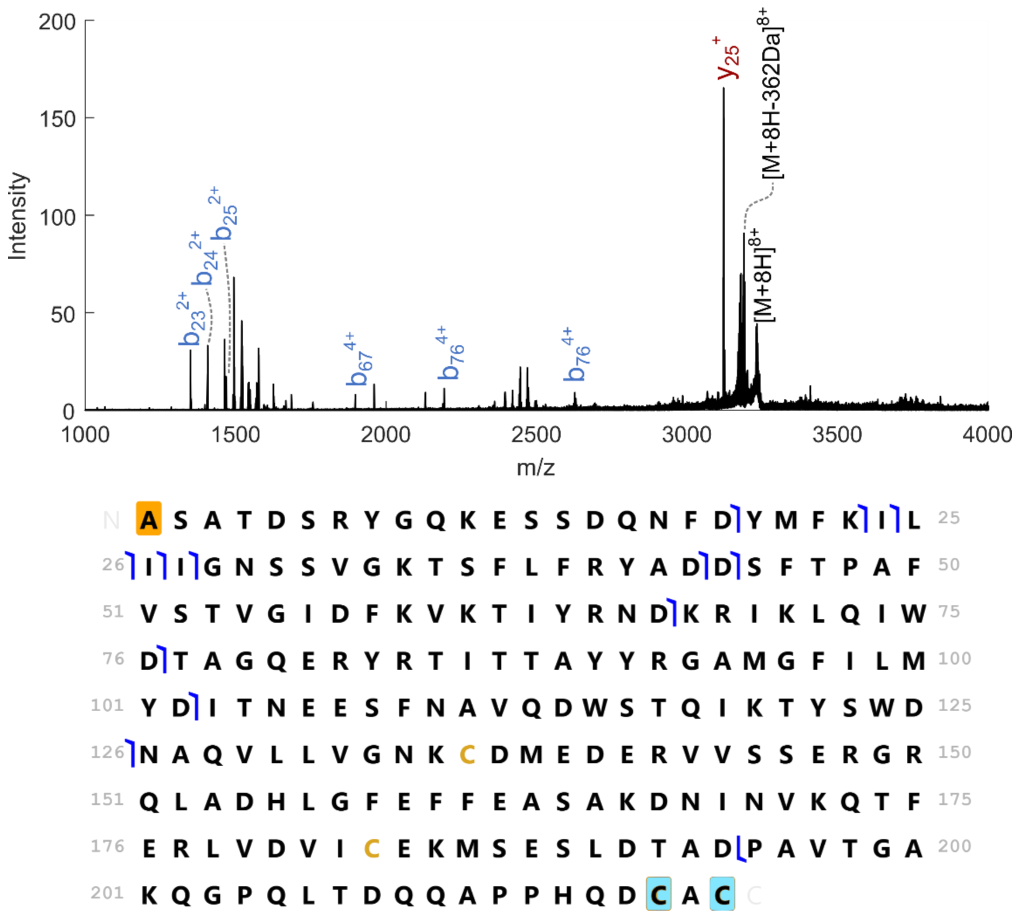

Figure S4: Top: Nano-DESI HCD MS^2 mass spectrum of m/z 3236.2 8+ ions of RAB3A (HCD 43% NCE). Bottom: Sequence coverage obtained. RAB3A was observed with two geranylgeranylation modifications, shown by the blue boxes, on the final two C-terminal cysteines. RAB3A also binds GDP non-covalently which is shown by the characteristic 362 Da loss from the parent ion.[2]

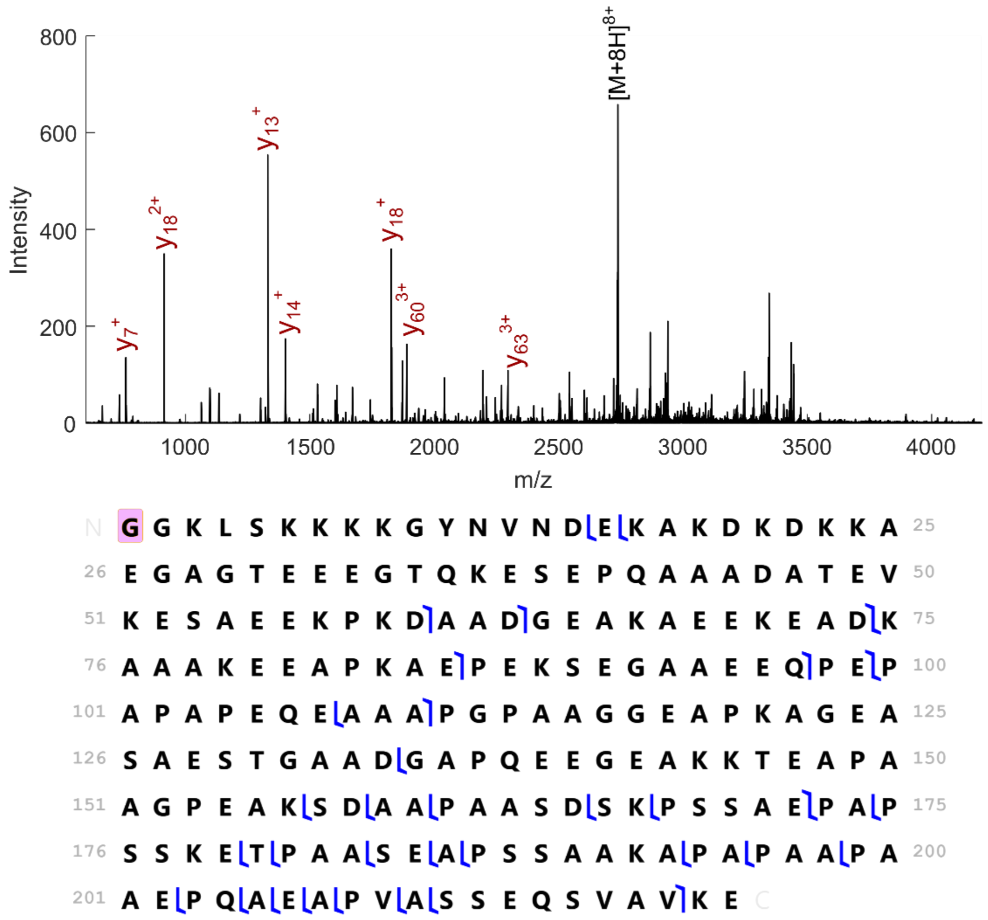

Figure S5: Top: Nano-DESI HCD MS^2 mass spectrum of m/z 2738.5 8+ ions of BASP1 (HCD 40% NCE). Bottom: Sequence coverage obtained. BASP1 was observed with an N-terminal myristoylation, marked by the pink box.

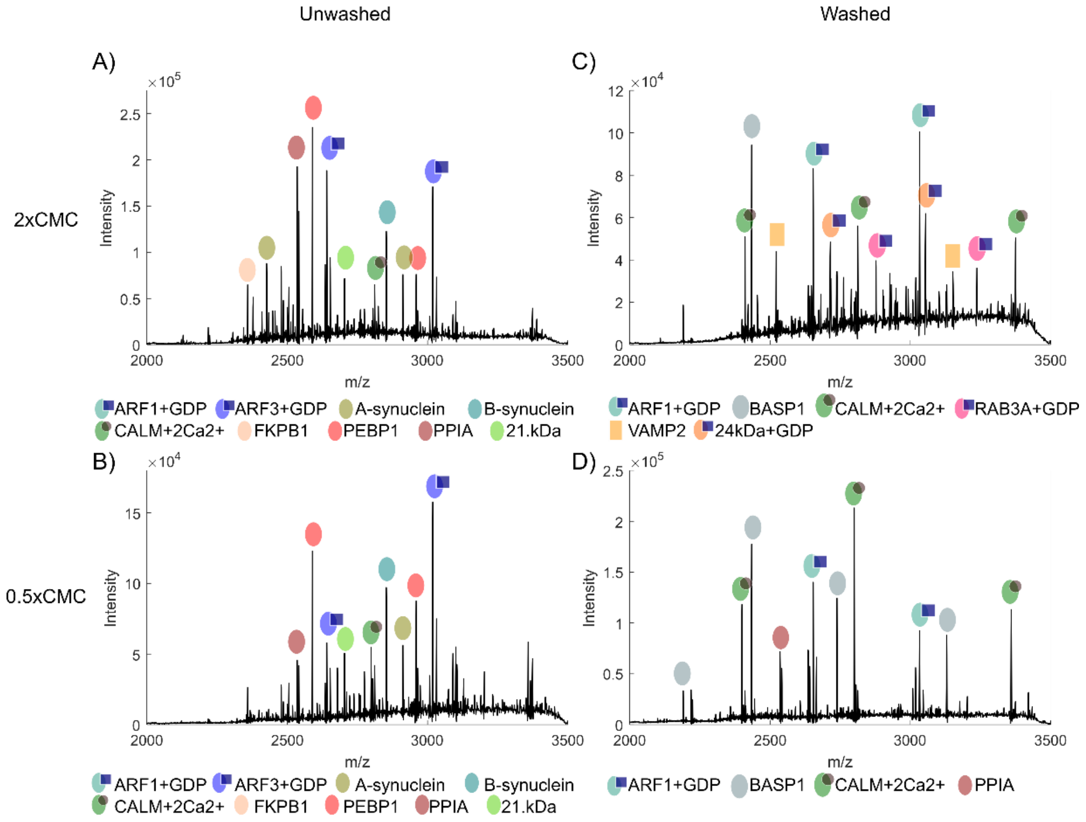

Figure S6: Representative nano-DESI mass spectra from the cortex of the brain obtained with a source compensation value (SCV) of 2.5% and a 1500 m/z SIM window centred around m/z 2750. A) Unwashed tissue sampled with 200 mM ammonium acetate containing 2x CMC C8E4, B) unwashed tissue sampled with 200 mM ammonium acetate containing 0.5x CMC C8E4, C) washed tissue sampled with 200 mM ammonium acetate containing 2x CMC C8E4 and D) washed tissue sampled with 200 mM ammonium acetate containing 0.5x CMC C8E4. Averaged mass spectra were achieved by recording three nano-DESI line scans. These were imported into MSI reader as an IMZL file where the ROI tool was used to export an averaged mass spectrum of the cortex.

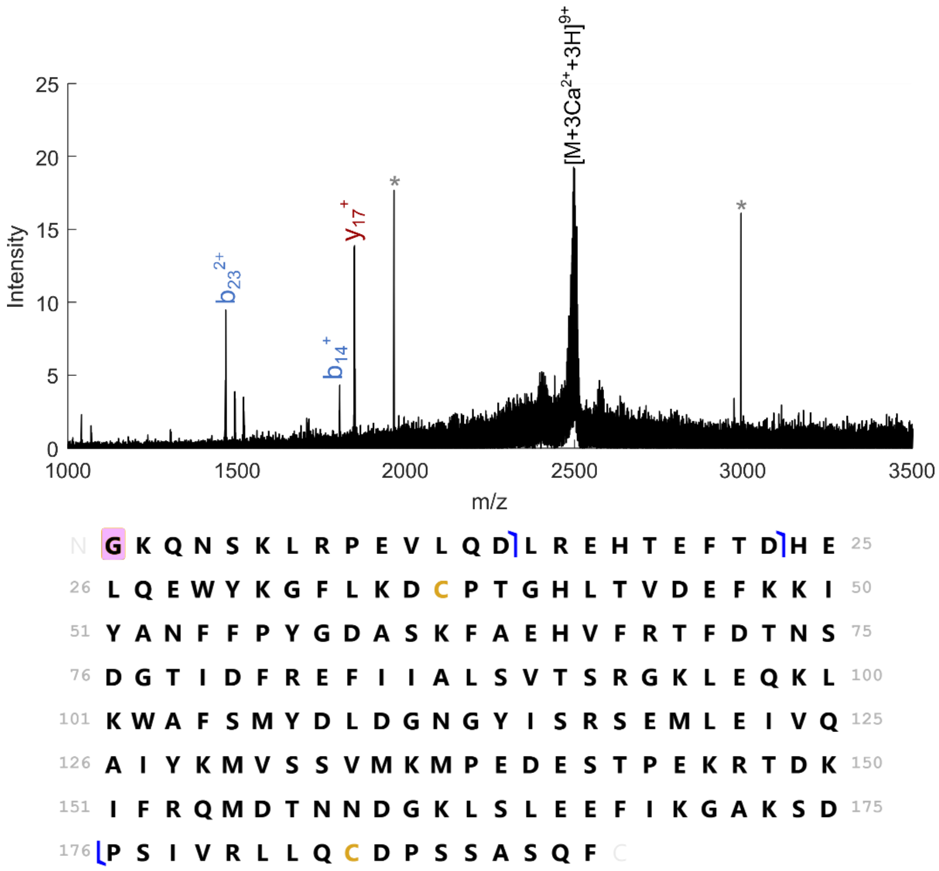

Figure S7: Top: Nano-DESI HCD MS^2 mass spectrum of m/z 2504.3 9+ ions of hippocalcin-like protein 1 (HCD 48% NCE). Bottom: Sequence coverage obtained. Hippocalcin-like protein was observed with an N-terminal myristoylation, marked by the pink box, and three Ca^{2+} ions bound.

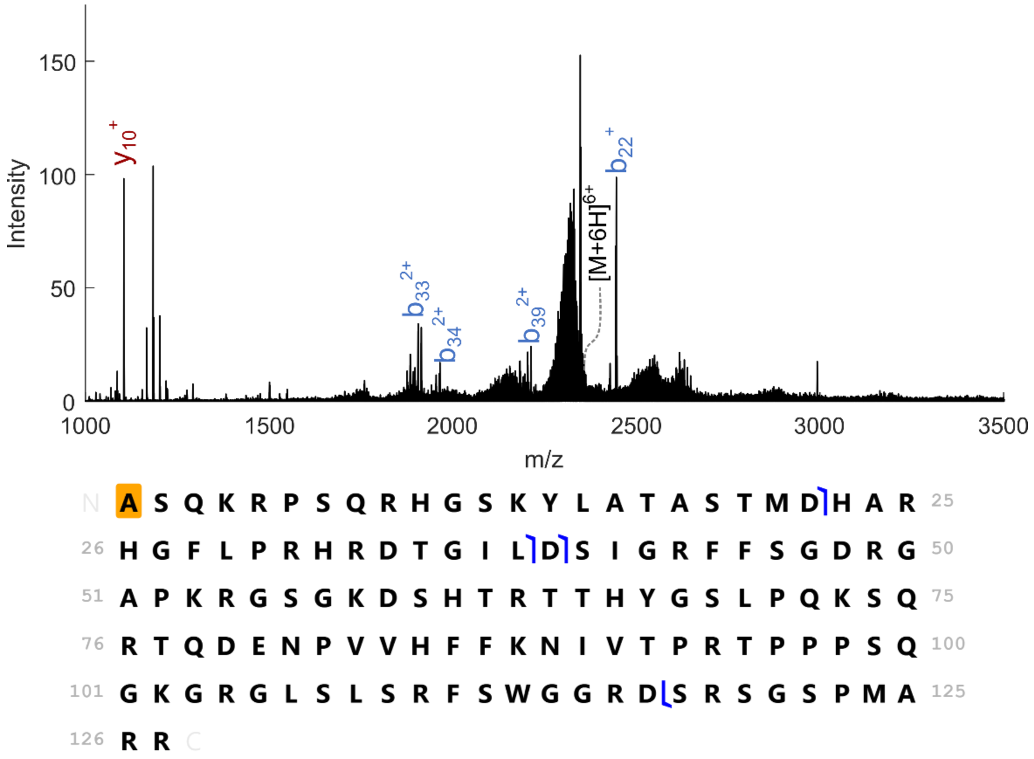

Figure S8: Top: Nano-DESI HCD MS^2 mass spectrum of m/z 2354.6 6+ ions of MBP short form (HCD 44% NCE). Bottom: Sequence coverage obtained. MBP has an N-terminal acetylation, marked by the orange box.

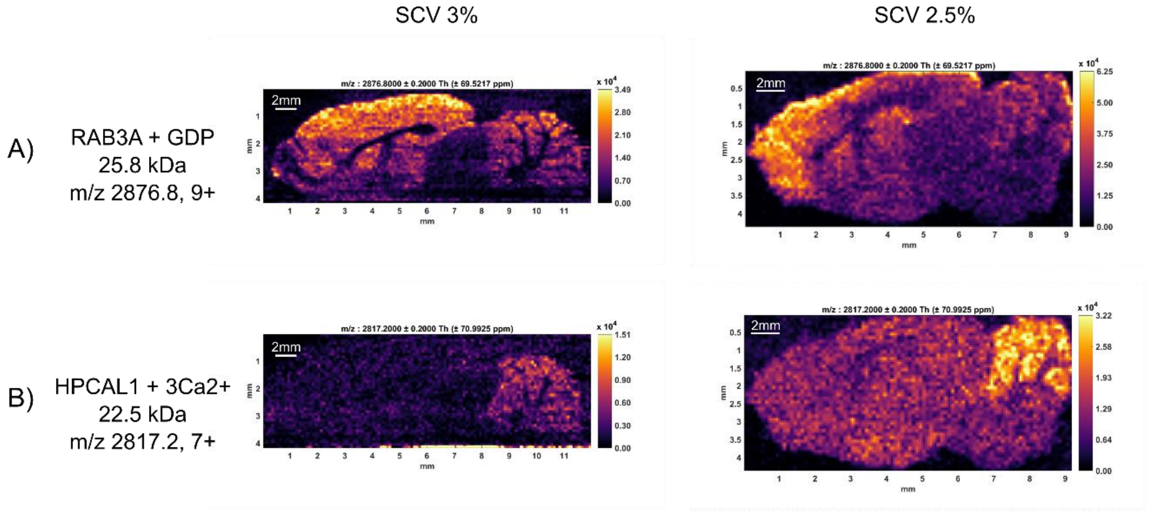

Figure S9: Single charge state ion images of A) Rab3A (m/z 2876.8), and B) HPCAL1 (m/z 2817.2), obtained with source compensation values of 3% and 2.5%,

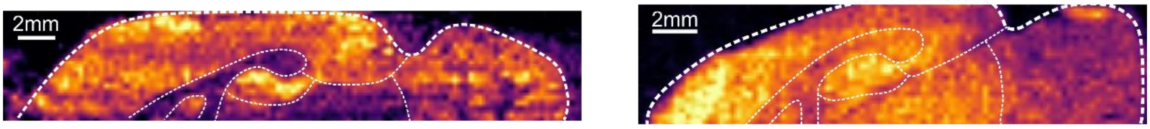

Figure S10: Ion images of [ARF1 + GDP] (m/z 2653.2; 8+) from unwashed (A) and washed (B) tissue sections. The spatial distribution of the ARF1 has been disrupted in the washed tissue.

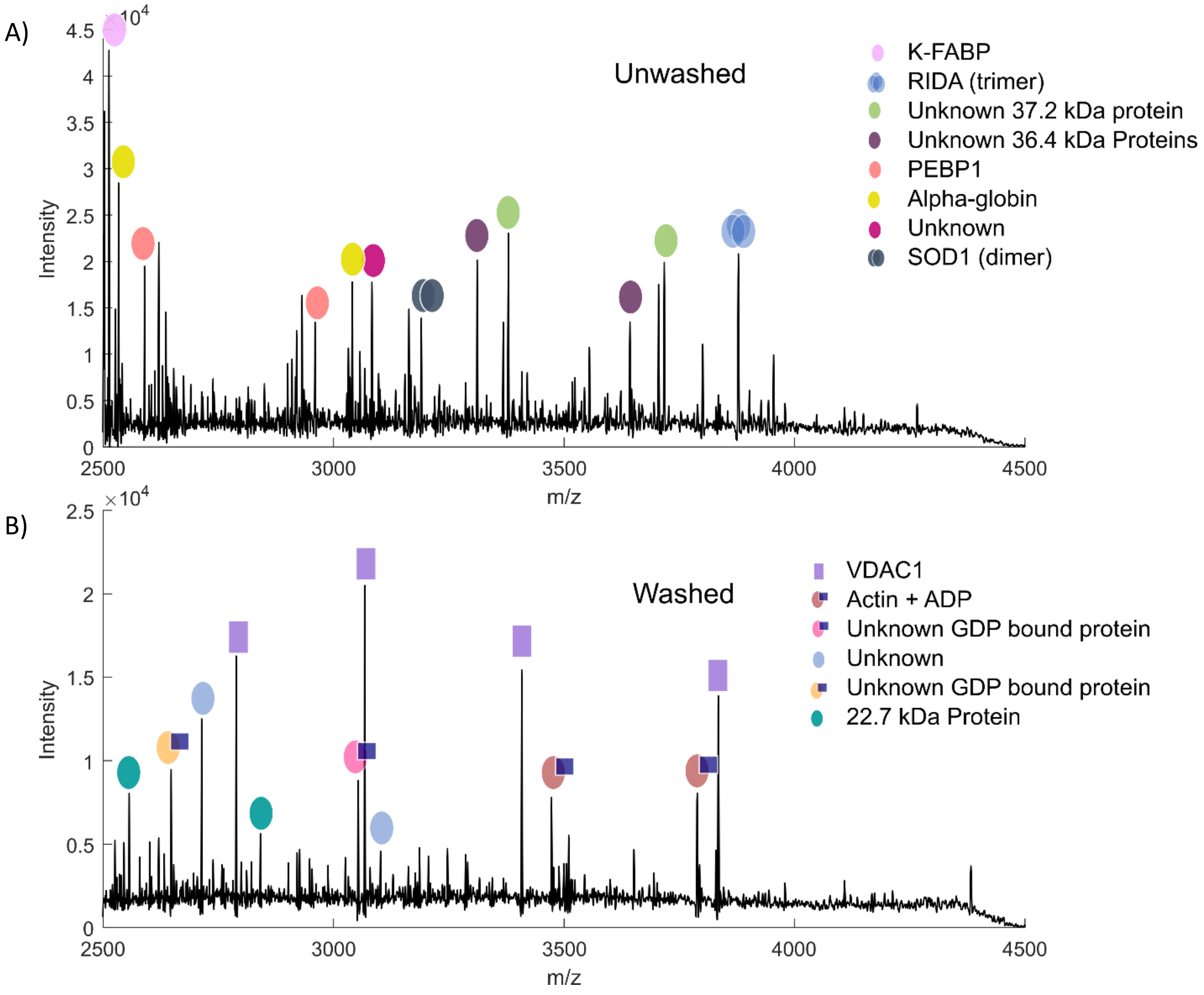

Figure S11: Representative nano-DESI mass spectra obtained from the cortex region of the kidney from A) unwashed and B) washed tissue sections. Sampling solvent comprised 200 mM ammonium acetate containing 2x CMC of detergent C8E4.

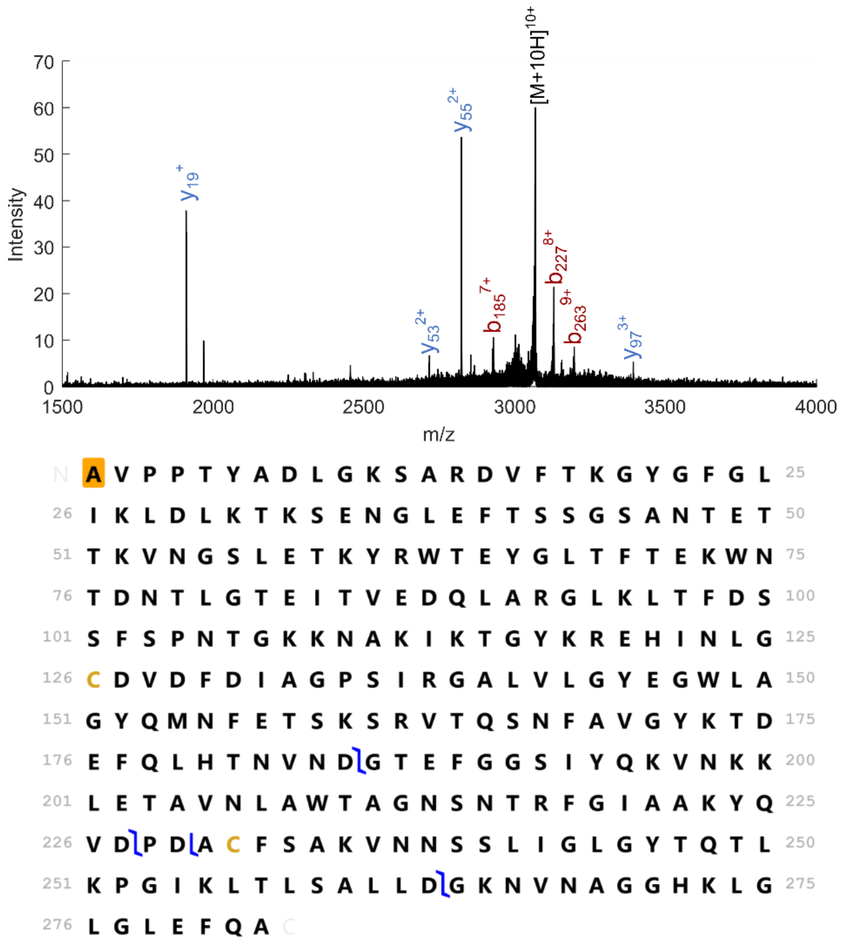

Figure S12: Top: Nano-DESI HCD MS^2 mass spectrum of m/z 3067.6 10+ ions of VDAC1 (HCD 55% NCE) from the cortex of rat kidney. Bottom: Sequence coverage obtained. VDAC1 has an N-terminal acetylation, marked by the orange box

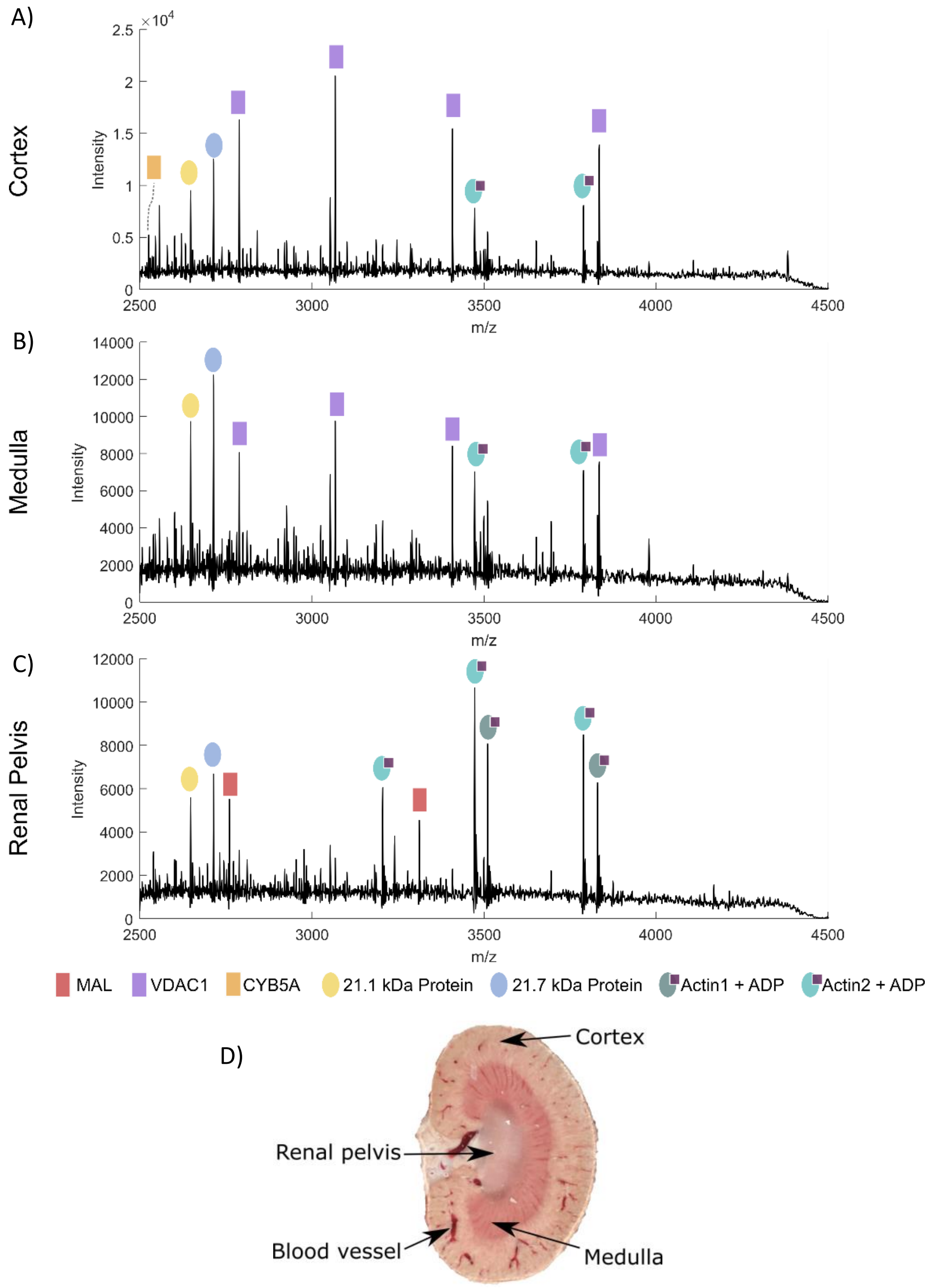

Figure S13: Representative nano-DESI mass spectra obtained from A) cortex, B) medulla, and C) renal pelvis regions of washed rat kidney section. Sampling solvent was 200 mM ammonium acetate + 2x CMC C8E4. D) Image showing the different regions of the kidney.

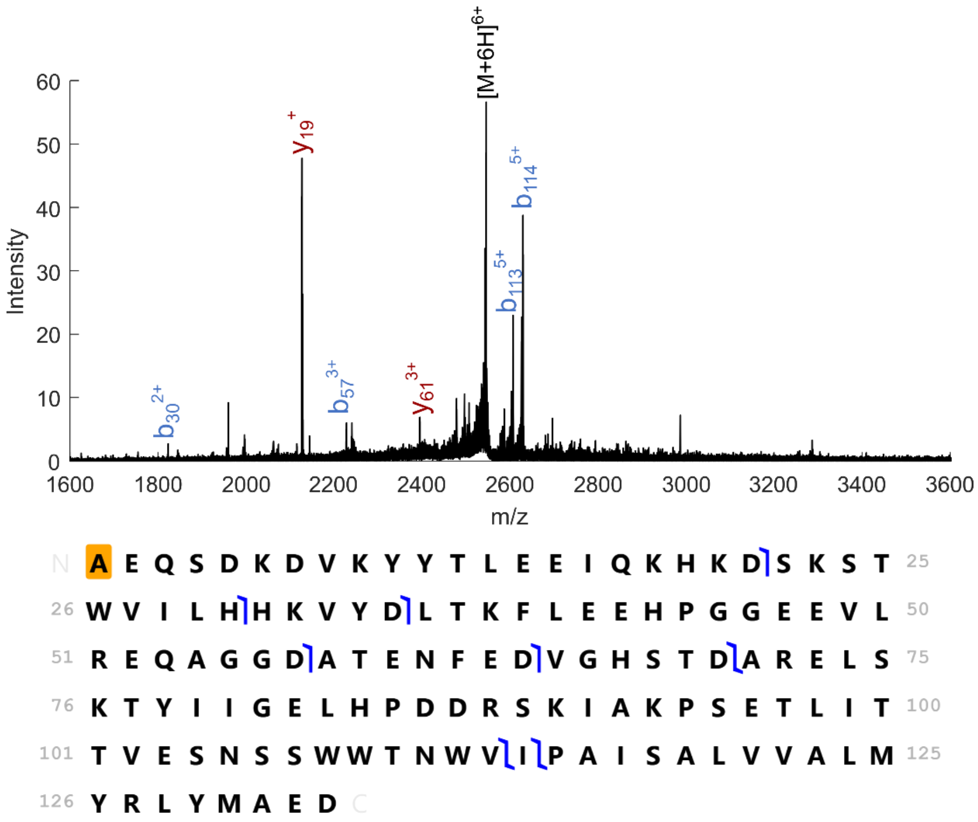

Figure S14: Top: Nano-DESI HCD MS^2 mass spectrum of m/z 2545.2 6+ ions of cytochrome B5 (HCD 48% NCE). Bottom: Sequence coverage obtained. Cytochrome B5 has an N-terminal acetylation marked by the orange box.

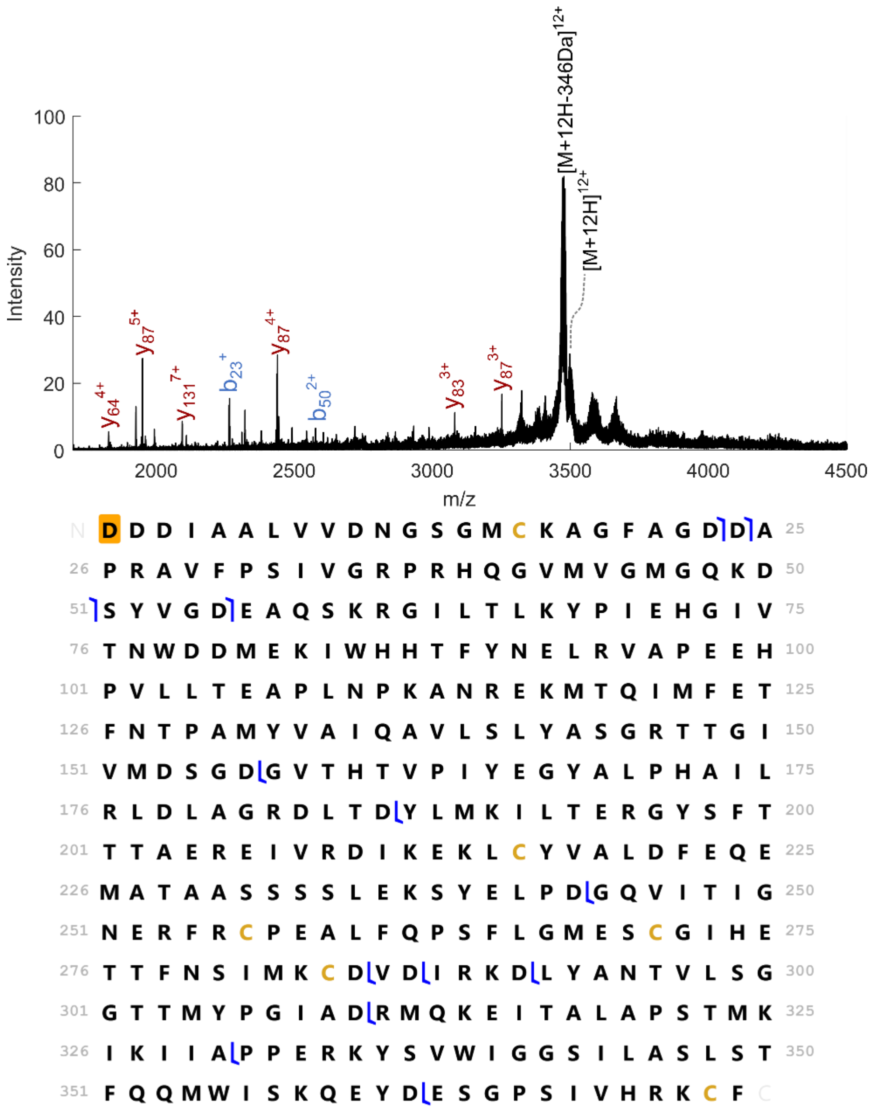

Figure S15: Top: Nano-DESI HCD MS^2 mass spectrum of m/z 3510.2 12+ ions of actin 1 with ADP bound (HCD 51% NCE). Bottom: Sequence coverage obtained. Actin 1 protein has an N-terminal acetylation marked by the orange box. Actin binds ADP non-covalently which is shown by the characteristic 346 Da loss from the parent ion.

References

1. Hale, O. J.; Hughes, J. W.; Sisley, E. K.; Cooper, H. J., Native Ambient Mass Spectrometry Enables Analysis of Intact Endogenous Protein Assemblies up to 145 kDa Directly from Tissue. *Analytical Chemistry* **2022,** *94* (14), 5608-5614.
2. Sisley, E. K.; Hale, O. J.; Styles, I. B.; Cooper, H. J., Native Ambient Mass Spectrometry Imaging of Ligand-Bound and Metal-Bound Proteins in Rat Brain. *Journal of the American Chemical Society* **2022,** *144* (5), 2120-2128.
3. Rajkumar, K.; Nichita, A.; Anoor, P. K.; Raju, S.; Singh, S. S.; Burgula, S., Understanding perspectives of signalling mechanisms regulating PEBP1 function. *Cell Biochemistry and Function* **2016,** *34* (6), 394-403.
4. Solà, C.; Barrón, S.; Tusell, J. M.; Serratosa, J., The Ca2+/calmodulin system in neuronal hyperexcitability. *The International Journal of Biochemistry & Cell Biology* **2001,** *33* (5), 439-455.
5. Huang, D.; Pei, Y.; Dai, C.; Huang, Y.; Chen, H.; Chen, X.; Zhang, X.; Lin, C.; Wang, H.; Zhang, R.; Wan, X.; Wang, L., Up-regulated ADP-Ribosylation factor 3 promotes breast cancer cell proliferation through the participation of FOXO1. *Experimental Cell Research* **2019,** *384* (2), 111624.
6. Suzuki, I.; Owada, Y.; Suzuki, R.; Yoshimoto, T.; Kondo, H., Localization of mRNAs for six ARFs (ADP-ribosylation factors) in the brain of developing and adult rats and changes in the expression in the hypoglossal nucleus after its axotomy. *Molecular Brain Research* **2001,** *88* (1), 124-134.
7. Boudhraa, Z.; Carmona, E.; Provencher, D.; Mes-Masson, A.-M., Ran GTPase: A Key Player in Tumor Progression and Metastasis. *Frontiers in Cell and Developmental Biology* **2020,** *8* (345).
8. Nasiri, E.; Sankowski, R.; Dietrich, H.; Oikonomidi, A.; Huerta, P. T.; Popp, J.; Al-Abed, Y.; Bacher, M., Key role of MIF-related neuroinflammation in neurodegeneration and cognitive impairment in Alzheimer's disease. *Mol Med* **2020,** *26* (1), 34.
9. Savaskan, N. E.; Fingerle-Rowson, G.; Buchfelder, M.; Eyüpoglu, I. Y., Brain Miffed by Macrophage Migration Inhibitory Factor. *International Journal of Cell Biology* **2012,** *2012*, 139573.
10. George, J. M., The synucleins. *Genome Biology* **2001,** *3* (1), reviews3002.1.
11. Hayashi, J.; Carver, J. A., β-Synuclein: An Enigmatic Protein with Diverse Functionality. *Biomolecules* **2022,** *12* (1).
12. Carnazza, K. E.; Komer, L. E.; Xie, Y. X.; Pineda, A.; Briano, J. A.; Gao, V.; Na, Y.; Ramlall, T.; Buchman, V. L.; Eliezer, D.; Sharma, M.; Burré, J., Synaptic vesicle binding of α-synuclein is modulated by β- and γ-synucleins. *Cell Reports* **2022,** *39* (2), 110675.
13. Nakajo, S.; Shioda, S.; Nakai, Y.; Nakaya, K., Localization of phosphoneuroprotein 14 (PNP 14) and its mRNA expression in rat brain determined by immunocytochemistry and in situ hybridization. *Molecular Brain Research* **1994,** *27* (1), 81-86.
14. Bendor, J. T.; Logan, T. P.; Edwards, R. H., The function of α-synuclein. *Neuron* **2013,** *79* (6), 1044-66.
15. Stefanis, L., α-Synuclein in Parkinson's disease. *Cold Spring Harb Perspect Med* **2012,** *2* (2), a009399.
16. Delcourt, V.; Franck, J.; Quanico, J.; Gimeno, J.-P.; Wisztorski, M.; Raffo-Romero, A.; Kobeissy, F.; Roucou, X.; Salzet, M.; Fournier, I., Spatially-Resolved Top-down Proteomics Bridged to MALDI MS Imaging Reveals the Molecular Physiome of Brain Regions. *Mol Cell Proteomics* **2018,** *17* (2), 357-372.
17. Ghandour, M. S.; Parkkila, A.-K.; Parkkila, S.; Waheed, A.; Sly, W. S., Mitochondrial Carbonic Anhydrase in the Nervous System. *Journal of Neurochemistry* **2000,** *75* (5), 2212-2220.
18. Tong, M.; Jiang, Y., FK506-Binding Proteins and Their Diverse Functions. *Curr Mol Pharmacol* **2015,** *9* (1), 48-65.
19. Schlüter, O. M.; Khvotchev, M.; Jahn, R.; Südhof, T. C., Localization Versus Function of Rab3 Proteins: EVIDENCE FOR A COMMON REGULATORY ROLE IN CONTROLLING FUSION*. *Journal of Biological Chemistry* **2002,** *277* (43), 40919-40929.

20. Wang, Y.; Sugita, S.; Südhof, T. C., The RIM/NIM Family of Neuronal C2 Domain Proteins: INTERACTIONS WITH Rab3 AND A NEW CLASS OF Src HOMOLOGY 3 DOMAIN PROTEINS*. *Journal of Biological Chemistry* **2000,** *275* (26), 20033-20044.
21. Camara, A. K. S.; Zhou, Y.; Wen, P.-C.; Tajkhorshid, E.; Kwok, W.-M., Mitochondrial VDAC1: A Key Gatekeeper as Potential Therapeutic Target. *Frontiers in Physiology* **2017,** *8*.
22. Koo, S. J.; Kochlamazashvili, G.; Rost, B.; Puchkov, D.; Gimber, N.; Lehmann, M.; Tadeus, G.; Schmoranzer, J.; Rosenmund, C.; Haucke, V.; Maritzen, T., Vesicular Synaptobrevin/VAMP2 Levels Guarded by AP180 Control Efficient Neurotransmission. *Neuron* **2015,** *88* (2), 330-44.
23. Raptis, A.; Torrejón-Escribano, B.; Gómez de Aranda, I.; Blasi, J., Distribution of synaptobrevin/VAMP 1 and 2 in rat brain. *J Chem Neuroanat* **2005,** *30* (4), 201-11.
24. Li, C.; Lim, S.; Braunewell, K. H.; Ames, J. B., Structure and Calcium Binding Properties of a Neuronal Calcium-Myristoyl Switch Protein, Visinin-Like Protein 3. *PLoS One* **2016,** *11* (11), e0165921.
25. Paterlini, M.; Revilla, V.; Grant, A. L.; Wisden, W., Expression of the neuronal calcium sensor protein family in the rat brain. *Neuroscience* **2000,** *99* (2), 205-216.
26. Wąsik, N.; Sokół, B.; Hołysz, M.; Mańko, W.; Juszkat, R.; Jagodziński, P. P.; Jankowski, R., Serum myelin basic protein as a marker of brain injury in aneurysmal subarachnoid haemorrhage. *Acta Neurochir (Wien)* **2020,** *162* (3), 545-552.
27. Guo, G.; Papanicolaou, M.; Demarais, N. J.; Wang, Z.; Schey, K. L.; Timpson, P.; Cox, T. R.; Grey, A. C., Automated annotation and visualisation of high-resolution spatial proteomic mass spectrometry imaging data using HIT-MAP. *Nature Communications* **2021,** *12* (1), 3241.
28. Hartl, M.; Puglisi, K.; Nist, A.; Raffeiner, P.; Bister, K., The brain acid-soluble protein 1 (BASP1) interferes with the oncogenic capacity of MYC and its binding to calmodulin. *Molecular Oncology* **2020,** *14* (3), 625-644.
29. Kashihara, M.; Miyata, S.; Kumanogoh, H.; Funatsu, N.; Matsunaga, W.; Kiyohara, T.; Sokawa, Y.; Maekawa, S., Changes in the localization of NAP-22, a calmodulin binding membrane protein, during the development of neuronal polarity. *Neuroscience Research* **2000,** *37* (4), 315-325.
30. Iino, S.; Kobayashi, S.; Maekawa, S., Immunohistochemical localization of a novel acidic calmodulin-binding protein, NAP-22, in the rat brain. *Neuroscience* **1999,** *91* (4), 1435-1444.
